# Association of body mass index with clinical outcomes for in-hospital cardiac arrest adult patients following extracorporeal cardiopulmonary resuscitation

**DOI:** 10.1371/journal.pone.0176143

**Published:** 2017-04-19

**Authors:** Eunmi Gil, Soo Jin Na, Jeong-Am Ryu, Dae-Sang Lee, Chi Ryang Chung, Yang Hyun Cho, Kyeongman Jeon, Kiick Sung, Gee Young Suh, Jeong Hoon Yang

**Affiliations:** 1 Department of Critical Care Medicine, Samsung Medical Center, Sungkyunkwan University School of Medicine, Seoul, Republic of Korea; 2 Department of Thoracic and Cardiovascular Surgery, Samsung Medical Center, Sungkyunkwan University School of Medicine, Seoul, Republic of Korea; 3 Division of Pulmonary and Critical Care Medicine, Department of Medicine, Samsung Medical Center, Sungkyunkwan University School of Medicine, Seoul, Korea; 4 Division of Cardiology, Department of Medicine, Samsung Medical Center, Sungkyunkwan University School of Medicine, Seoul, Republic of Korea; Azienda Ospedaliero Universitaria Careggi, ITALY

## Abstract

**Background:**

Obesity might be associated with disturbance of cannulation in situation of extracorporeal cardiopulmonary resuscitation (ECPR). However, limited data are available on obesity in the setting of ECPR. Therefore, we investigated the association between body mass index (BMI) and clinical outcome in patients underwent ECPR.

**Methods:**

From January 2004 to December 2013, in-hospital cardiac arrest patients who had ECPR were enrolled from a single-center registry. We divided patients into four group according to BMI defined with the WHO classification (underweight, BMI < 18.5, n = 14; normal weight, BMI = 18.5–24.9, n = 118; overweight, BMI = 25.0–29.9, n = 53; obese, BMI ≥ 30, n = 15). The primary outcome was survival to hospital discharge.

**Results:**

Analysis was carried out for a total of 200 adult patients (39.5% females). Their median BMI was 23.20 (interquartile range, 20.93–25.80). The rate of survival to hospital discharge was 31.0%. There was no significant difference in survival to hospital discharge among the four groups (underweight, 35.7%; normal, 31.4%; overweight, 30.2%; obese, 26.7%, *p* = 0.958). Neurologic outcomes (*p* = 0.85) and procedural complications (*p* = 0.40) were not significantly different among the four groups either. SOFA score, initial arrest rhythm, and CPR to extracorporeal membrane oxygenation (ECMO) pump on time were significant predictors for survival to discharge, but not BMI.

**Conclusion:**

BMI was not associated with in-hospital mortality who underwent ECPR. Neurologic outcomes at discharge or procedural complications following ECPR were not related with BMI either.

## Introduction

Obesity has been implicated as one of the major risk factors for cardiovascular disease and congestive heart failure (HF) [1]. However, evidence from clinical cohorts indicates an obesity paradox in overweight and obese patients who seem to have more favorable short-term and long-term prognosis than leaner patients [2,3]. On the other hand, in cases of cardiac arrest, relationship with body mass index (BMI) and mortality is not clearly defined. Some studies suggest that increased BMI is positive factor on survival [4–6], while recent data suggest that obesity is related with higher mortality [7].

Patients with cardiac arrest generally have poor prognosis [8,9]. Recently, Chen et al. [10,11] have reported that extracorporeal cardiopulmonary resuscitation (ECPR) is superior to conventional cardiopulmonary resuscitation (CPR) for in-hospital cardiac arrest patients. Although the association of obesity with clinical outcome in patients who underwent conventional CPR has been studied in a small number of previous studies up to date [5,6], clinical impact of obesity in a setting of ECPR has not been evaluated. Therefore, the objective of this study was to assess the association between body mass index (BMI) and clinical outcomes after in-hospital cardiac arrest following ECPR.

## Methods

### Study population

**Fig 1:**
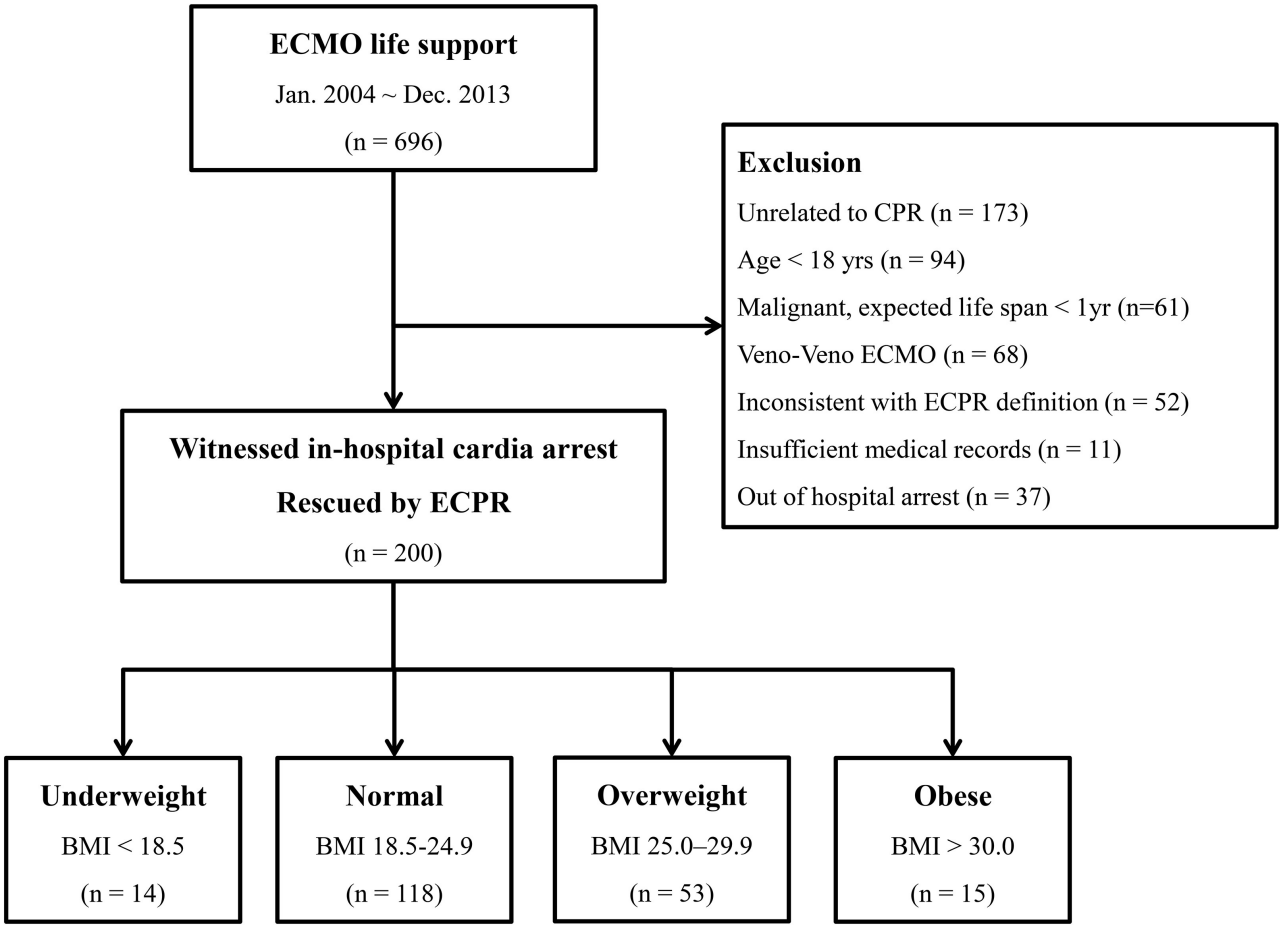
Scheme of group distribution in the registry.

This was a retrospective, single-center, and observational study of consecutive adult patients with in-hospital cardiac arrest who had ECPR at Samsung Medical Center between January 2004 and December 2013. This study was approved by the Samsung Medical Center Institutional Review Board (IRB No. 2016-05-086). Informed consent requirement was waived due to its retrospective nature. As described in detail previous study [12], clinical, laboratory, and outcome data were collected by a trained study coordinator using a standardized case report form. Additional information was obtained by reviewing hospital records. We included 696 consecutive patients who underwent extracorporeal membrane oxygenation (ECMO) life support during the study period. Of these patients, those unrelated to CPR, under 18 years of age, with malignancy and expected life span of less than 1 year, with veno-venous ECMO, inconsistent with ECPR definition, with insufficient medical records, or with out-of-hospital arrest were excluded. Finally, a total of 200 patients with witnessed cardiac arrest rescued by veno-arterial ECMO life support were eligible for this study (Fig 1).

### Definition and outcomes

BMI was defined as weight (in kilograms) divided by the square of height (in meters) (kg/m^2^). It was used to categorize patients based on the World Health Organization’s definition [13]: Underweight (BMI < 18.5), Normal range (BMI 18.5–24.9), Overweight (BMI 25–29.9), and Obese (BMI ≥ 30).

ECMO was considered for patients who underwent prolonged CPR (>10 min) without sustained return of spontaneous circulation (ROSC). Definition of sustained ROSC was continuous maintenance of spontaneous circulation for ≥ 20 min. In this study, ECPR was defined as an intention-to-treat with hemodynamic ECMO support during cardiac massage regardless of interim ROSC [14]. CPR to ECMO pump-on time was defined as the time from initiation of cardiac massage to the time of ECMO pump-on. Survival after veno-artiral ECMO (SAVE) score was measured before ECMO cannulation. And post-ECMO initial Sequential Organ Failure Assessment (SOFA) score was measured for all patients using the worst value of each scoring item within 24 h of the event. The primary outcome was survival to hospital discharge. The secondary outcomes were good neurological outcomes defined as cerebral performance categories (CPC) scale 1 or 2 and ECMO related complications.

### Procedure

CPR was led by the CPR team of the hospital. Request call for ECPR was up to the CPR team leader when CPR was performed for more than 10 minutes or for unstable vital signs or recurrent cardiac arrest. The final decision to institute ECMO and the ECMO cannulation procedure during CPR was determined by ECMO specialists such as interventional cardiologists and cardiac surgeons. Our ECMO cannulation and management flow was described in a previous report [12]. Arterial cannulae sized 14 to 21 French and venous cannulae sized 21 to 28 French were used. After successful ECMO pump-on, in order to achieve an ideal cardiac index greater than 2.2 L/min/body surface area (m^2^), central mixed venous saturation over 70%, and mean arterial pressure over 65 mm Hg, the initial revolutions per minute of the ECMO device were adjusted based upon the above criteria.

### Statistical analysis

Continuous variables were expressed as medians and interquartile ranges (IQRs). Comparisons for continuous variables were made using t-test or Wilcoxon rank-sum test when applicable. Categorical data were tested using chi-squared test. A multivariate logistic regression model was used to identify predictors of survival to discharge. All variables associated with survival discharge were analyzed by univariate analysis. Variables with p value of less than 0.2 were considered clinically relevant were included in the multivariate analysis. All tests were two-tailed and a *p*-value < 0.05 was considered as statistically significant. Statistical tests were performed using SPSS software (SPSS Inc., Chicago IL, USA) version 22.0 for Windows.

## Results

### Baseline and procedural characteristics

Demographic characteristics of patients are summarized in Table 1. Their median age was 62.5 years old (interquartile range [IQR], 51.0–74.0 years). Of the 200 patients, 79 (39.5%) were females. Their median BMI was 23.20 (IQR, 20.93–25.80). The majority of patients (n = 118, 59%) were normal weight, 14 (7%) were underweight, 53 (26.5%) were overweight, and 15 (7.5%) were obese. There were no significant differences in demographic characteristics among the four groups except for age. The initial arrest and procedural findings are shown in Table 2. A cardiogenic origin was the most common cause of cardiopulmonary resuscitation (n = 154, 77.0%). Pulseless electrical activity was the most common first monitored arrest rhythm (n = 113, 56.5%). ICU was the most common location of ECPR (n = 100, 50%), and 51 patients (25.5%) were supported by ECMO at catheterization laboratory. Another 11 patients (5.5%) had at general ward, 26 (13.0%) at emergency department, and 12 (6.0%) at operating room. ROSC before ECMO pump-on occurred in 69 patients (34.5%). The median CPR to ECMO pump-on time was 35.0 minutes (IQR, 22.0–55.0 min). All patients supported with mechanical ventilator, 73 patients (36.5%) supported with CRRT, 31 patients (15.5%) supported with intra-aortic balloon pump, and 151 patients (75.5%) needed vasopressor support. There were also no significant differences in initial arrest and procedural findings among the four groups.

**Table 1:** Baseline patient characteristic.

	Underweight (n = 14)	Normal (n = 118)	Overweight (n = 53)	Obese (n = 15)	p-value
**Age, yrs**	36.50 (18–89)	63.50 (19–86)	62.00 (21–83)	65.00 (19–86)	0.002
**Gender (female)**	7 (50)	41 (34.7)	21 (39.6)	10 (66.7)	0.10
**BMI, kg/m2**	16.40 (15.1–18.4)	21.70 (18.5–24.9)	26.50 (25.0–29.6)	31.20 (30.0–35.3)	< 0.001
**Comorbidities**					
** Diabetes**	5 (35.7)	59 (50)	23 (43.4)	6 (40.0)	0.64
** Hypertension**	4 (28.6)	55 (46.6)	27 (50.9)	8 (53.3)	0.48
** Dyslipidemia**	0	16 (13.6)	9 (17.0)	3 (20.0)	0.37
** Current smoker**	3 (21.4)	31 (26.3)	9 (17.0)	1 (6.7)	0.25
** Chronic kidney disease**	1 (7.1)	19 (16.1)	6 (11.3)	1 (6.7)	0.58
** Peripheral vascular disease**	1 (7.1)	7 (5.9)	4 (7.5)	0	0.75
** Prior myocardial infarction**	0	20 (16.9)	6 (11.3)	2 (13.3)	0.33
** Previous stroke**	1 (7.1)	20 (16.9)	5 (9.4)	2 (13.3)	0.51
** SAVE score**	-9 (-15.0–-2.0)	-9 (-13.0–-6.8)	-10 (-12.0–-7.5)	-10 (-12.0–-8.0)	0.88

BMI, body mass index; SAVE, survival after veno-arterial extracorporeal membrane oxygenation

Values are n (%) or median (Interquartile range).

**Table 2:** Arrest and procedural characteristics.

	Underweight (n = 14)	Normal (n = 118)	Overweight (n = 53)	Obese (n = 15)	p-value
**Arrest cause**					0.99
** Cardiogenic origin**	11 (78.6)	90 (76.3)	41 (77.4)	12 (80.0)	
** Sepsis**	1 (7.1)	12 (10.2)	5 (9.4)	1 (6.7)	
** Hypovolemia**	0	12 (10.2)	6 (11.3)	1 (6.7)	
** Respiratory deterioration**	2 (14.3)	1 (0.8)	0	0	
** Neurogenic origin**	0	1 (0.8)	0	0	
** Unknown**	0	2 (1.7)	1 (1.9)	1 (6.7)	
**First monitored arrest rhythm**					0.29
** Asystole**	3 (21.4)	17 (14.4)	9 (17.0)	2 (13.3)	
** PEA**	7 (50.0)	61 (51.7)	34 (64.2)	11 (73.3)	
** VF/Pulseless VT**	4 (28.6)	40 (33.9)	10 (18.9)	2 (13.3)	
**Location of ECMO cannulation**					0.40
** Intensive Care Unit**	8 (57.1)	56 (47.5)	32 (60.4)	4 (26.7)	
** Catheterization laboratory**	4 (28.6)	29 (24.6)	11 (20.8)	7 (46.7)	
** General ward**	1 (7.1)	7 (5.9)	2 (3.8)	1 (6.7)	
** Emergency department**	1 (7.1)	17 (14.4)	6 (11.3)	2 (13.3)	
** Operating room**	0	9 (7.6)	2 (3.8)	1 (6.7)	
**ROSC before ECMO pump on**	5 (35.7)	44 (37.3)	16 (30.2)	4 (26.7)	0.74
**CPR to ECMO pump on time (min)**	42 (16–75)	35 (8–89)	35 (5–84)	35 (5–91)	0.46
**CRRT**	4 (28.6)	42 (35.6)	23 (43.4)	4 (26.7)	0.55
**IABP**	1 (7.1)	23 (19.5)	7 (13.2)	0	0.16
**Vasopressor support**	11 (78.6)	94 (79.7)	38 (71.7)	8 (53.3)	0.13
**Initial SOFA score after ECPR**	12.00 (5–16)	13.00 (6–19)	14.00 (5–20)	12.00 (10–18)	0.10

BMI, body mass index; PEA, pulseless electrical activity; VF, ventricular fibrillation; VT, ventricular tachycardia; ROSC, return of spontaneous circulation; ECMO, extracorporeal membrane oxygenation; CPR, cardiopulmonary resuscitation; CRRT, continuous renal replacement therapy; IABP, intra-aortic balloon pump; SOFA, Sequential Organ Failure Assessment; ECPR, ECMO-assisted cardiopulmonary resuscitation

Values are n (%) or median (Interquartile range).

### Clinical outcomes

Clinical outcomes following ECPR are summarized in Table 3 and Fig 1. Among the 200 adult cardiac arrest patients who underwent ECPR, successful ECMO weaning was achieved in 86 (43.0%) patients [underweight, 6 (42.9%); normal weight, 51 (43.2%); overweight, 23 (43.4%); obese, 6 (40.0%); *p* = 0.98]. Survival to hospital discharge was achieved in 62 (31.0%) patients [underweight, 5 (35.7%); normal weight, 37 (31.4%); overweight, 16 (30.2%); obese, 4 (26.7%); *p* = 0.96]. Good neurological outcomes at discharge (CPC scale 1 or 2) were achieved in 52 (26.0%) patients [underweight, 4 (28.6%); normal weight, 28 (23.7%); overweight, 16 (30.2%); obese, 4 (26.7%); *p* = 0.85]. And among these, 50 patients maintained as CPC scale 1 or 2 at 12 months follow-up. Failure of ECMO initiation due to cannulation failure occurred in 7 (3.5%) patients [underweight, 0; normal weight, 5 (4.2%); overweight, 2 (3.8%); obese, 0; *p* = 0.74]. A total of 30 (15.0%) patients [underweight, 0; normal weight, 18 (15.3%); overweight, 9 (17.0%); obese, 3 (20.0%); *p* = 0.40] suffered from procedure related complications, including 22 cases of groin hematoma, 18 cases of limb ischemia, 11 cases of cannulation site bleeding, 15 cases of gastrointestinal bleeding, and 7 cases of procedure-related infection.

**Table 3:** Extracorporeal cardiopulmonary resuscitation outcomes.

	Underweight (n = 14)	Normal (n = 118)	Overweight (n = 53)	Obese (n = 15)	p-value
**Survival to discharge**	5 (35.7)	37 (31.4)	16 (30.2)	4 (26.7)	0.96
**Good neurologic outcomes at discharge** ^a^	4 (28.6)	28 (23.7)	16 (30.2)	4 (26.7)	0.85
**Successful initiation of ECMO**	14 (100)	111 (94.1)	48 (90.6)	15 (100)	0.39
**Successful weaning**	6 (42.9)	51 (43.2)	23 (43.4)	6 (40.0)	0.98
**Procedure related complications**	0	18 (15.3)	9 (17.0)	3 (20.0)	0.40
**Groin hematoma**	0	10 (8.5)	9 (17.0)	2 (13.3)	0.40
**Limb ischemia**	0	11 (9.3)	5 (9.4)	2 (13.3)	0.63
**Cannulation site bleeding**	0	7 (5.9)	3 (5.7)	1 (6.7)	0.83
**GI bleeding**	0	13 (11.0)	2 (3.8)	0	0.13
**Procedure-related infections**	0	6 (5.1)	1 (1.9)	0	0.51

BMI, body mass index; ECMO, extracorporeal membrane oxygenation; GI, gastrointestinal

^a^Cerebral performance categories scale 1 or 2

Values are n (%).

### Predictors of survival to discharge

Binary logistic regression analysis was performed to recognize predictors of survival to discharge. Significant univariate predictors of survival to discharge were SOFA score following ECPR, arrest rhythm such as pulseless electrical activity and ventricular arrhythmia, and CPR to pump-on time. In multivariate binary logistic regression analysis, independent predictors for the occurrence of survival to discharge were also SOFA score following ECPR, arrest rhythm such as pulseless electrical activity and ventricular arrhythmia, and CPR to pump-on time, but not BMI (Table 4).

**Table 4:** Risk factors for survival to hospital discharge.

	Univariate		Multivariate	
	OR (95% CI)	p-value	OR (95% CI)	p-value^a^
**Age**	0.99 (0.97–1.01)	0.339	0.98 (0.96–1.00)	0.083
**CPR at daytime**	1.54 (0.81–2.95)	0.189		
**SAVE score before ECPR**	1.06 (0.99–1.13)	0.097		
**Location of ECMO cannulation**		0.351		
** ICU**	1			
** Cardiac catheterization laboratory**	1.19 (0.59–2.40)			
** General ward**	0.20 (0.03–1.63)			
** Emergency department**	0.74 (0.28–1.93)			
** Operating room**	0.40 (0.08–1.93)			
**SOFA score after ECPR**	0.74 (0.63–0.87)	<0.001	0.67 (0.55–0.81)	<0.001
**initial ECG rhythm**		0.005		<0.001
** Asystole**	1		1	
** PEA**	4.02 (1.14–14.12)	0.030	6.16 (1.58–24.05)	0.009
** VF/Pulseless VT**	7.78 (2.11–28.64)	0.002	17.86 (4.15–76.81)	<0.001
**CPR to ECMO pump-on time (per minute)**	0.98 (0.96–0.99)	0.005	0.97 (0.95–0.99)	<0.001
**BMI**		0.951		0.946
** Underweight**	1.17 (0.37–3.73)	0.791	1.01 (0.26–3.92)	0.992
** Normal**	1		1	
** Overweight**	0.91 (0.45–1.84)	0.793	1.27 (0.54–2.96)	0.59
** Obese**	0.77 (0.23–2.56)	0.665	0.90 (0.23–3.45)	0.872

SOFA, Sequential Organ Failure Assessment; ECG, electrocardiography; PEA, pulseless electrical activity; VF, ventricular fibrillation; VT, ventricular tachycardia; CPR, cardiopulmonary resuscitation; ECMO, extracorporeal membrane oxygenation; BMI, body mass index

^a^Adjusted by Age, SOFA score, initial ECG rhythm, CPR to ECMO pump-on time and BMI.

## Discussion

In this study, we determined the association between BMI and survival to discharge after ECPR for in-hospital cardiac arrest adult patients in a single center registry from January 2004 to December 2013. Increased BMI was not associated with in-hospital mortality who underwent ECPR. Neurologic outcomes at discharge or procedural complications following ECPR were not related with BMI either. Initial SOFA score, initial ECG rhythm, and CPR to ECMO pump-on time were significant predictors for survival to discharge, but not BMI.

In the general population, a relationship between obesity and increased mortality rate has been reported [15,16]. Elevated BMI is also a risk factor of sudden death [17]. The most common causes of sudden death in patients with morbid obesity are eccentric and concentric cardiac hypertrophy and coronary heart disease which is less frequently [18]. However, evidence from clinical cohorts indicates an obesity paradox in overweight and obese patients who seem to have a more favorable prognosis in cardiovascular disease patients [2,3]. And obesity paradox was observed in critically ill patients in Korea [19].

However, in case of cardiac arrest, relationship with BMI and mortality is not clearly defined. White et al. [20] have reported survival was not related to body weight in patients with out-of-hospital cardiac arrest. And another study from Geri et al. [7] have reported obesity was associated with higher 30 day mortality. Theoretically, obese survivors of cardiac arrest should have worse outcomes than non-obese patients considering resuscitation challenges. It might be more difficult to resuscitate obese patients due to difficulties in providing adequate chest compressions, ventilation, and oxygenation. However, recent studies have shown better outcomes for overweight and obese patients after cardiac arrest. Jain et al.[4] have reported overweight patients had higher rates of survival to discharge in cases of cardiac arrest with shockable rhythms. Bunch et al. [5] have reported a higher survival for overweight and obese patients compared to that for patients with BMI less than 25.0 kg/m^2^ after witnessed out-of-hospital cardiac arrest due to ventricular fibrillation. Testori et al. [6] have also found that patients with moderately elevated BMI have better neurological prognosis, although BMI might have no direct influence on six-month survival. Chen et al. [10,11] have reported that ECPR is superior to conventional CPR for in-hospital cardiac arrest patients. Although the association of obesity with clinical outcome in the setting of conventional CPR has been studied in a small number of previous studies, to date, data on the clinical impact of obesity on clinical outcomes in patients who underwent ECPR are unavailable. Therefore, we determined the impact of BMI on clinical outcomes in patients who underwent ECPR from single-center registry and found that obesity was not associated with in-hospital mortality or procedural complications following ECPR.

In a previous large-scale study, an important decision for implementation of ECMO in patients with in-hospital cardiac arrest has been suggested to be the separation of non-shockable rhythm from VF/pulseless VT [21]. The initial severity of organ failure has been found to be independent risk factors for in-hospital death [21]. Similarly, in our study, initial arrest rhythm and SOFA score are independent prognostic factors for survival to discharge. Therefore, decisions regarding the implementation of ECMO during CPR should be made carefully while considering many factors such as initial arrest rhythm, severity of organ failure, and CPR duration. Generally, it is difficult to access central vascular lines of obese patients. They tend to have more mechanical complications during vascular line access which could lead to prolonged CPR to ECMO pump-on time due to cannulation difficulties. As a result, obesity could be considered as a risk factor for poor outcome of ECPR. However, our study showed that there were no significant differences in CPR to ECMO pump-on time, cannulation failure, or procedure related complications among four groups of patients. Our findings suggest that obesity should not be regarded as a contraindication to initiation of ECPR.

Our study had several limitations. First, there might be potential risk of confounding variables because it was not a randomized control trial. Second, we used a limited number of patients which limited our conclusions. Recent published study demonstrated that obese group has higher incidences of diabetes, hypertension, dyslipidemia, and prior history of cardiac or cerebral problems [22]. However, in our study, there are no statistical differences of diabetes, hypertension, and VT/VF. These findings of our study might be caused by a small number of subjects. Thus, future studies with larger cohorts are needed. In addition, only 15 patients had BMI above 30 kg/m^2^ and no one had BMI of more than 40 kg/m^2^, which limited our conclusions regarding higher BMI values. Markedly low prevalence of obesity in our registry may possibly to genetic characteristics as well as socio-economic variables. Thus, our findings are difficult to generalize to Western population. Third, body size was measured upon admission at ICU. This might have reduced the impact of fluid overload on BMI determination. Fourth, waist circumference and waist-to-hip ratio as another parameters of obesity were unavailable in our registry. Finally, we did not have any information on important outcome variables such as CPR quality or post arrest management in our ECMO registry.

## Conclusion

BMI was not associated with in-hospital mortality who underwent ECPR. Neurologic outcomes at discharge or procedural complications following ECPR were not related with BMI either.

## Supporting information

S1 Data Data of ECPR after in-hospital cardiac arrest. (XLSX)
